# Knowledge loss of medical students on first year basic science courses at the university of Saskatchewan

**DOI:** 10.1186/1472-6920-6-5

**Published:** 2006-01-14

**Authors:** Marcel F D'Eon

**Affiliations:** 1 Educational Support and Development, College of Medicine, University of Saskatchewan, B103 Health Sciences Building, 107 Wiggins Raod, Saskatoon, Saskatchewan, S7N 5E5, Canada

## Abstract

**Background:**

Many senior undergraduate students from the University of Saskatchewan indicated informally that they did not remember much from their first year courses and wondered why we were teaching content that did not seem relevant to later clinical work or studies. To determine the extent of the problem a course evaluation study that measured the knowledge loss of medical students on selected first year courses was conducted.

This study replicates previous memory decrement studies with three first year medicine basic science courses, something that was not found in the literature. It was expected that some courses would show more and some courses would show less knowledge loss.

**Methods:**

In the spring of 2004 over 20 students were recruited to retake questions from three first year courses: Immunology, physiology, and neuroanatomy. Student scores on the selected questions at the time of the final examination in May 2003 (the 'test') were compared with their scores on the questions 10 or 11 months later (the 're-test') using paired samples *t *-tests. A repeated-measures MANOVA was used to compare the test and re-test scores among the three courses. The re-test scores were matched with the overall student ratings of the courses and the student scores on the May 2003 examinations.

**Results:**

A statistically significant main effect of knowledge loss (F = 297.385; p < .001) and an interaction effect by course (F = 46.081; p < .001) were found. The students' scores in the Immunology course dropped 13.1%, 46.5% in Neuroanatomy, and 16.1% in physiology. Bonferroni *post hoc *comparisons showed a significant difference between Neuroanatomy and Physiology (mean difference of 10.7, p = .004).

**Conclusion:**

There was considerable knowledge loss among medical students in the three basic science courses tested and this loss was not uniform across courses. Knowledge loss does not seem to be related to the marks on the final examination or the assessment of course quality by the students.

## Background

With all the time and money spent teaching medical students one must wonder how well that investment is paying off. What portion are they retaining in memory? What are they learning? – This seems to be the central question for medical education [1]. Surely if students are not remembering what they have been taught then the effort was wasted; if students cannot make use of the knowledge they have been taught, if that knowledge becomes inert and inaccessible, then why teach it in the first place [2-4]. This question is important for both pre-clinical and clinical courses. This study set out to determine to what extent students enrolled in our program were remembering (and forgetting) what they had initially learned in some of their first year basic science courses as a step towards making important curricular decisions [5,6].

The University of Saskatchewan undergraduate program in medicine is currently organized into departmentally based and inter-departmental courses. In the first year following a minimum of two years of pre-medical university training, students take a variety of basic science courses along with clinical courses in basic history taking, communications, physical examination skills, ethics, professional issues, and patient centred practice. Approximately half of the class is female and the average age is in the mid twenties. Administrators at the medical school were most concerned about the retention rate in the basic sciences: gross anatomy, histology and cell biology, physiology, neuroanatomy, immunology, and biochemistry. Many of our students in later years indicated informally that they did not remember much from first year and wondered why we were teaching some things that did not seem relevant to later studies or clinical work. Frequent comments on course evaluations also led us wonder if students were retaining the information long enough to be of some use to them in later training. The decision was made to determine the extent of the problem by conducting a course evaluation study by measuring the knowledge loss of medical students in selected first year courses.

This study is an extension of previous work examining retention of knowledge and memory decay. Much of the early work was completed some time ago and recent studies were not found. Swanson [7] used the USLME Part 1, primarily a basic science examination, as their reference point. They tested the same group of students approximately 15 months later with a basic science test and discovered a decline of 2.9 percentage points on average between the scores of the two tests. Compared to the knowledge loss reported in other studies, a loss of 2.9% is quite small and reason to celebrate. However, the USMLE examines only important aspects of basic science knowledge taught in medical school and is not a clear standard for the retention of actual lecture material or course content that is typically crowded with the teacher's favourite material [8].

For example Watt [9] found a decline of 21.5% on pre-clinical knowledge of oral biology when the same test was administered 20 month later to dental students. Krebs [10] discovered that medical students retained only 65% of the simple basic science knowledge. Whatever method of instruction is used [11,12] and whatever types of questions [13] were examined the findings point to a loss of knowledge. The factor that seemed to make a difference is reinforcement over time [14] where scores improved slightly 21 months after the end of the course.

The study set out to replicate other memory decrement studies not with one course but with three first year medicine basic science courses, something that did not show up in the literature. It was expected that some courses would show more and some courses would show less knowledge loss. Furthermore, some courses that changed from one year to the next might show different retention rates. Perhaps this information could be used over time for course and curriculum evaluation purposes.

## Methods

In late March of 2004 29 (second year) students were recruited to retake 20 examination questions from the Immunology course offered in the second term of the first year. In early April another 25 students (many of the same ones who came in March for Immunology) were recruited to retake 21 questions from their full year physiology course and 25 questions from the neuroanatomy course given in the second term of their first year. Students were not told on which courses they would be re-tested but we were assured by the students that even if we had told them no one would have studied! They were enticed to participate with the promise of free lunch which they gladly accepted.

The instructors of the three courses selected the questions for the re-test; the faculty were not blinded to the purpose of the study. Therefore, where I had the data, student scores on the selected questions on the re-test were compared with their examination score using paired samples *t *-test to ensure that the selected questions were representative of the examination as a whole. The final examination scores of the volunteers were also compared with those of the whole class using independent samples *t *-tests to ensure that the students who came to take the re-test were representative of the whole class.

Student scores on the questions at the time of the final examination in May 2003 (the 'test') were compared with their scores on the questions 10 or 11 months later (the 're-test') using paired samples *t *-tests. For the physiology course the specific scores on the selected questions were not available so the final examination scores in total were used as a proxy for the 'test' scores and compared to the scores for the specific 21 re-test questions for the re-test takers.

This study also compared the knowledge loss for all three courses together since the hypothesis was that there would be differences in knowledge loss among the courses tested. A repeated-measures MANOVA was used to compare the test and re-test scores for the three courses.

Since differences among courses were expected related perhaps to course approval ratings and initial examination results for the three courses both the course evaluations and test results for the courses were compared to re-test results.

## Results

### In general

**Figure 1:**
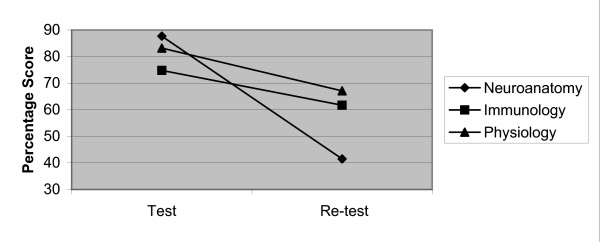
Test and Re-test scores 10 or 11 months apart for three basic science courses at the University of Saskatchewan.

Using a repeated measures MANOVA a statistically significant main effect of knowledge loss (F = 297.385; p < .001) and an interaction effect by course (F = 46.081; p < .001) were found. The students' scores in the Immunology course dropped 13.1%, 46.5% in Neuroanatomy, and 16.1% in their physiology course. There was also a difference between courses. Bonferroni *post hoc *comparisons showed a significant difference between Neuroanatomy and Physiology (mean difference of 10.7, p = .004). For a summary of the test and re-test scores, examination scores, and knowledge decrement for all three courses please see Table 1. Figure 1 shows this same analysis in graphic form.

### Immunology

For the Immunology course there was no difference between the sampled 20 questions and the examination results for the student volunteers with means of 74.8% and 77.0% respectively. The correlation between these scores was, as expected, very high at .910 (p < .001). Using an independent samples t-test no significant difference between the examination scores of the students who volunteered to do the re-test compared to those who did not was found: they were 77.0% and 72.3% respectively. This established that the selected questions were representative of the examination and that those who volunteered to take the retest were representative of the entire class.

The paired samples *t *-test for test and re-test on the 20 immunology questions for the student volunteers revealed a statistically significant difference (t = -5.852; p < .001) with means of 74.8% and 61.7%. This showed a relative knowledge decrement of 17.6% over the ten months. There was a moderately high correlation between the scores on the 20 questions on the original examination in May 2003 and the re-test in April 2004 of .619 (p < .001).

### Neuroanatomy

For the neuroanatomy course there was a statistically significant difference between the 25 questions sampled and the examination results in May 2003 for the student volunteers *t *= -4.466, p < .001, means of 87.7% and 82.5% respectively. The correlation between the 25 selected questions and the examination results was high at .820 (p < .001) for the student volunteers. The scores on the selected questions were higher than the scores on the original examination itself and those who did well on the examination also had high marks on the selected 25 questions. The questions sampled were not representative of the examination from May 2003; they were in fact much easier than most of the questions on the examination.

There was a significant difference between the scores on the 25 questions at examination time (87.7%) and the re-test scores 11 months late (41.5%) (*t *= 17.427, p < .001) and a very weak, negligible correlation (.310, p = .140). This was a relative knowledge decrement of over 52%.

### Physiology

Scores for the student volunteers on the selected questions from the final physiology examination were not available. Based on the results from the other two courses showing congruence between the examination results and the selected re-test question the May 2003 examination scores were used as an approximation of the scores on the 21 selected questions and used as the 'test' scores. The examination results of those who volunteered to take the retest were compared with the results of those who did not volunteer and no differences were found. When the examination scores (83.2%) were compared with the scores on the 21 retest questions (67.1%) using a paired samples *t *-test a statistically significant difference (*t *= 5.121, p < .001) was found. Those scores were moderately correlated (r = .523, p = .007). The relative knowledge loss was 19.4%.

## Discussion

### Knowledge loss confirmed

In each of the three individual courses studied there was a statistically significant difference between the test and the re-test results. In both immunology and physiology the magnitude of the loss was consistent with what most researchers have found [7-12] and in neuroanatomy the drop was quite dramatic (52.7% relative knowledge loss). The knowledge loss in this neuroanatomy course is all the more notable since the questions selected were actually easier than the entire original examination. Even greater knowledge loss might have been discovered if the questions had been representative of the test as a whole. Not only are there differences between test and re-test scores on all courses in this study but there are differences among courses for the same students over the same time period. Post-hoc analysis showed a statistically significant difference between two of the individual courses (neuroanatomy and physiology). Since the same students experienced different magnitudes of knowledge loss in different courses in the same year then clearly the problem is not only the learner forgetting but the course and or the program not teaching! If the problem was intrinsic to medical students then we would expect to see similar results across courses but instead we see large differences indicating that to some extent the variance is found in the courses and/or the curriculum.

### Reasons for knowledge loss

Previous studies indicate that the performance of medical (and dental) students on written tests of knowledge generally declines over time and that this probably varies with ongoing reinforcement and the quality of initial learning. Halpern [15] states that strong long term memory has been associated with over-learning in the initial phase and the spacing of learning over time. The study by Giles [11] noted that learning of visually presented material was initially better but after four months there was no difference when compared to verbally presented material. Certainly initial learning is necessary for long term retention but evidently not sufficient. This is confirmed by Conway [3] who identified factors in long term retention of information at the post-secondary level: taking successive courses (overlearning and reinforcement), initial 'active' learning (though not necessarily high marks on courses), and the nature of the material (procedural over declarative and general over specific). Bransford, Brown, and Cocking [16] note that the elements of courses that promote initial learning are a focus on meaning and understanding rather than memorization, adequate time to learn especially complex material, and deliberate effective engagement with the task (practice). This means that cramming is counter-productive in the long-term although in the short term it may produce high grades on examinations [12].

A well organized curriculum can facilitate review and over-learning of key concepts as more advanced courses and clinical experiences explicitly and intentionally use and build on earlier learning. Baddeley [17], Regehr and Norman [18], and Gardiner [19] have much to say about the importance of the characteristics of practice and reinforcement including distributed, expanding, elaborative rehearsal centred on recall, not recognition or re-presentation of content. Since both initial learning and spaced practice are necessary for long-term memory, knowledge loss cannot be attributed solely to the weakness of the course but must be shared with the program deficiency that knowledge is not reinforced over time.

It is likely that the neuroanatomy course content received the least amount of review and spaced practice. By the time the students took the retest on the three basic science courses they had not yet begun their study of neurology. Immunology, on the other hand, would have been somewhat reinforced in several locations in the curriculum (a 5-week course on hematology and a full year course on clinical microbiology). Similarly, physiology content would have been reviewed in multiple places throughout the second year. The more dramatic knowledge loss in neuroanatomy may be due to both poor initial learning and lack of reinforcement.

Another factor to consider is the quality of the examination both technically and clinically. It is possible that the questions were ambiguous or otherwise poorly constructed. It is also possible that the questions might be asking for clinically irrelevant knowledge and no amount of relevant clinical courses or experiences would provide much opportunity for reinforcement.

### Predicting knowledge loss

First, the original scores on the selected re-test questions seem to be unrelated to the amount of knowledge loss as noted in Conway *et al *[3]. The score on the neuroanatomy course test questions was 87.7% (much higher than any of the other two courses studied) matched with a much greater relative knowledge drop (52%). The low and statistically insignificant correlation between the test and re-test scores (.310, p = .140) indicates that for individuals initial high examination scores did not match in any way the scores on the re-test. For immunology, on the other hand, the score on the re-test question was initially the lowest but the correlation between test and re-test scores for individuals was moderately high at .619 (p < .001) and the relative knowledge loss (17.6%) was the least of the three courses tested. Certainly the readers may recall courses where high marks reflected a high degree of learning of core principles that would then be translated into greater long term retention. One can also recall from personal experience courses where some serious cramming resulted in a strong showing on the examination followed rapidly by knowledge loss, a form of bulimic learning [8]. Drawing on past experience, published research [3], and this small sample I would suggest that a score on an original examination may not predict knowledge loss on any given course. Educators and administrators should be suspicious that the examination and course content might not be clinically relevant if the marks on the original examination were not correlated to re-test scores.

Second, were student evaluations might be somehow related to or predictive of knowledge loss? Given that knowledge loss is multifactorial (initial learning and ongoing rehearsal) it is unlikely that student evaluations (able only to approximate course quality and therefore perhaps initial learning) would be good predictors of long term retention. The overall rating of the neuroanatomy course (60% approval) is slightly lower than immunology (68%) and much lower than physiology (75%). There was virtually no difference between the knowledge losses for the immunology and physiology courses but seemed to be as much of a difference in approval ratings as between neuroanatomy and immunology. (Statistical tests to determine these relationships were not applied since the raw data from the course evaluations was not available.)

There is no clear pattern in these relationships and further research is required to explore whatever link there may be between knowledge loss and examination results and student evaluations of courses.

## Conclusion

There was considerable knowledge loss among medical students in the three basic science courses tested at the University of Saskatchewan and this loss was not uniform across courses. In some courses students forget more and in others they forget less. This phenomenon does not seem to be related to the marks on the final examination or to the evaluation of course quality by the students.

Courses that exhibit greater knowledge decrement will have generated either less initial learning or will have been reinforced less over the intervening months or both. At a local level awareness of knowledge loss can be used to target courses for review and revision and identify weaknesses in the overall educational program. Perhaps, as other researchers [13] have suggested, scores for some courses might actually improve as the knowledge is practiced and used over time. Maximizing knowledge improvement over time might become a goal of curriculum planners rather than the more negative goal of minimizing knowledge loss. Further research is required to help educators easily identify knowledge loss and the factors that contributed to that knowledge loss.

## Competing interests

The author(s) declare that they have no competing interests.

## Pre-publication history

The pre-publication history for this paper can be accessed here:



## Figures and Tables

**Table 1:** Comparing the scores on tests and re-tests of knowledge for three basic science courses

Course	Exam %	Test % on selected questions	Re-test % on selected questions	Relative Knowledge Loss^1^	Course Evaluation	Correlation between Test re-test scores
Neuroanatomy N = 24	82.5	87.7	41.5	52.7	3.6/6.0 (60%)	.310 p = .140
Immunology N = 29	77.0	74.8	61.7	17.6	4.1/6.0 (68%)	.619 p < .001
Physiology N = 25	83.2	Not available	67.1	19.4^2^	4.5/6.0 (75%)	.523 p = .007

^1^Calculated as a percentage of the original examination score on the selected re-test questions.

^2^Calculated using the original examination mark as an estimate of test score on selected questions.

## References

[B1] ten Cate O, Snell L, Mann K, Vermunt J (2004). Orienting teaching toward the learning process. Acad Med.

[B2] Cox K (1987). Knowledge which cannot be used is useless. Med Teacher.

[B3] Conway MA, Cohen G, Stanhope N (1992). Very long-term memory for knowledge acquired at school and university. App Cognitive Psych.

[B4] Ellis JS, Semb GB, Cole B (1998). Very long-term memory for information taught in school. Contemp Educ Psych.

[B5] Harden RM (1986). Approaches to curriculum planning. Med Education.

[B6] Sanson-Fisher R, Rolfe I (2000). The content of undergraduate health professional courses: a topic largely ignored?. Med Teacher.

[B7] Swanson DB, Case SM, Luecht RM, Dillon GF (1996). Retention of basic science information by fourth year medical students. Acad Med.

[B8] Nelson CE (2001). What is the most difficult step we must take to become great teachers?. The Nat Teach and Lear Forum.

[B9] Watt ME (1987). Retention of preclinical knowledge by clinical students. Med Education.

[B10] Krebs R, Hofer R, Bloch R, Guibert J-J (1994). Conversation et oubli des connaissances en biologie acquises pour le premier examen propédeutique de medicine. MEDUCS Bulletin de l'Association Suisse d'Education Medicale.

[B11] Harrison A (1995). Using knowledge decrement to compare medical students' long term retention of self-study and lecture materials. Assess and Eval in Higher Educ.

[B12] Sissons JC, Swartz RD, Wolf FM (1992). Learning, retention and recall of clinical information. Med Education.

[B13] Giles RM, Johnson MR, Knight KE, Zammett S, Weinman J (1982). Recall of lecture information: a question of what, when, and where. Med Education.

[B14] Blunt MJ, Blizard PJ (1975). Recall and retrieval of anatomical knowledge. Br J of Med Educ.

[B15] Halpern FH (2003). Thought and Knowledge: An introduction to critical thinking.

[B16] Bransford JD, Brown AL, Cocking RR, editors (2000). How People Learn: Brain, Mind, Experience, and School.

[B17] Baddeley A (1998). Human Memory: Theory and Practice.

[B18] Regehr G, Norman GR (1998). Issues in cognitive psychology: implications for professional education. Acad Med.

[B19] Gardiner JM, Gawlik B, Richardson-Klavehn A (1994). Maintenance rehearsal affects knowing, not remembering; elaborative rehearsal affects remembering, not knowing. Psych Bulletin & Rev.

